# The Contribution of the Ion–Ion and Ion–Solvent Interactions in a Molecular Thermodynamic Treatment of Electrolyte Solutions

**DOI:** 10.1021/acs.jpcb.2c03915

**Published:** 2022-11-17

**Authors:** Spiros Kournopoulos, Mirella Simões Santos, Srikanth Ravipati, Andrew J. Haslam, George Jackson, Ioannis G. Economou, Amparo Galindo

**Affiliations:** † Department of Chemical Engineering, Sargent Centre for Process Systems Engineering, and Institute for Molecular Science and Engineering, Imperial College, London, London SW7 2AZ, United Kingdom; ‡ Laboratoire de Chimie, École Normale Supérieure de Lyon, 46 Allée d’Italie, 69364 Lyon, France; § Australian Institute for Bioengineering and Nanotechnology, The University of Queensland, Brisbane, Queensland 4072, Australia; ∥ Chemical Engineering Program, Texas A&M University at Qatar, Doha 23874, Qatar

## Abstract

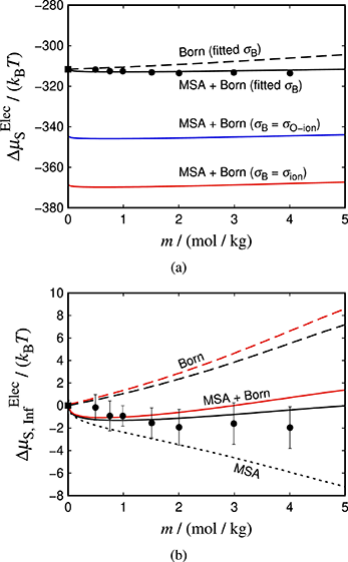

Developing molecular equations of state to treat electrolyte solutions is challenging due to the long-range nature of the Coulombic interactions. Seminal approaches commonly used are the mean spherical approximation (MSA) and the Debye–Hückel (DH) theory to account for ion–ion interactions and, often, the Born theory of solvation for ion–solvent interactions. We investigate the accuracy of the MSA and DH approaches using each to calculate the contribution of the ion–ion interactions to the chemical potential of NaCl in water, comparing these with newly computer-generated simulation data; the ion–ion contribution is isolated by selecting an appropriate primitive model with a Lennard-Jones force field to describe the solvent. A study of mixtures with different concentrations and ionic strengths reveals that the calculations from both MSA and DH theories are of similar accuracy, with the MSA approach resulting in marginally better agreement with the simulation data. We also demonstrate that the Born theory provides a good qualitative description of the contribution of the ion–solvent interactions; we employ an explicitly polar water model in these simulations. Quantitative agreement up to moderate salt concentrations and across the relevant range of temperature is achieved by adjusting the Born radius using simulation data of the free energy of solvation. We compute the radial and orientational distribution functions of the systems, thereby providing further insight on the differences observed between the theory and simulation. We thus provide rigorous benchmarks for use of the MSA, DH, and Born theories as perturbation approaches, which will be of value for improving existing models of electrolyte solutions, especially in the context of equations of state.

## 1 Introduction

An accurate description of the thermodynamics of electrolyte solutions is of critical importance in many industrial applications. Characteristic examples include enhanced-oil-recovery, the purification of pharmaceuticals, and the chemical design of batteries and supercapacitors in the energy industry, amongst others. Unfortunately, the long-range nature of the Coulombic and polar interactions significantly influence thermodynamic properties of electrolyte solutions, making statistical-mechanical descriptions of these systems especially difficult. Moreover, it is very challenging to develop models that are suitable for the many different applications of electrolytes, retaining good performance across the wide range of relevant conditions of temperature, pressure, concentration, ionic strength, and electrolyte and solvent type.

In the current work, we focus on thermodynamic approaches for the estimation of the Helmholtz free energy of electrolyte solutions; in particular, we consider the extensions of statistical associating fluid theory (SAFT).^1,2^ Through the differentiation of the Helmholtz free energy, it is possible to obtain all of the equilibrium thermodynamic properties of interest.^3^ It has been shown^4,5^ that equations of state (EoS) for electrolyte solutions can be developed by using a reference model that accounts for the short-range (repulsive and dispersive) interactions accurately. Electrostatic contributions are then added as perturbation terms to account for Coulombic interactions resulting from ion–ion interactions (the ion term) and ion–solvent interactions (the Born term). Several authors^4,6−12^ have developed and parametrized SAFT extensions to describe the thermodynamics of aqueous electrolyte solutions following this premise.

Coulombic contributions arising from ion–ion interactions are typically treated by implementing one of two classical theories: either the Debye–Hückel (DH)^13^ or the Mean Spherical Approximation (MSA).^14^ In the DH theory,^13^ the linear form of the Poisson–Boltzmann (PB) equation is solved to describe the thermodynamics of low-concentration strong electrolyte solutions. By contrast, in the MSA theory,^14,15^ the Ornstein–Zernike equation is solved using a closure for which it is assumed that the potential of mean force is equal to its low-density limit (the pair potential). Wei and Blum^16^ further developed the MSA theory to account explicitly for solvent–ion interactions. Many studies have included an investigation of the relative merits of the DH and MSA approaches. For example, it has been shown^17−19^ that MSA allows for a better description of thermodynamic properties of solutions of strong electrolytes, such as vapor pressure, density, and ionic activity coefficients. In a study that is particularly pertinent in the context of the current work, Maribo-Morgensen et al.^19^ state that, in practice, there is little difference in the performance of the DH and MSA approaches. These authors regressed parameters for electrolyte solution models using DH and MSA and showed that the difference in performance of the two approaches is not significant, in the sense that the additional error associated with the DH theory can be easily absorbed by adjusting the parameters of the model.

While there are a number of studies^20−31^ that have focused on developing force fields to model the thermodynamic properties of electrolyte solutions, there have been comparatively fewer studies in which authors have attempted to benchmark theories for electrolyte solutions. In this regard, the work of Valleau and co-workers^32,33^ who conducted Monte Carlo simulations of model electrolyte solutions, benchmarking several theoretical approaches including DH and MSA, is of great significance. Specifically, they focused on the concentration dependence of the activity and osmotic coefficients, the average potential energy, and the radial distribution functions of the ions. Based on their calculations, they showed that both DH and MSA lead to reasonable agreement in terms of the activity coefficient, osmotic coefficient, and average potential energy. However, it was also shown that these theories cannot be used to model the radial distribution function at moderate or high salt concentrations due to the neglect of the higher-order many-body interaction terms. It was shown that quantitative agreement can be achieved with neither theory, unless some modification is introduced.^34−37^ Although the work of Valleau and co-workers is of critical importance to understanding how calculations from the different theories can be compared with simulation data, its focus was on finding the best possible theoretical approach for modeling ion–ion interactions regardless of computational complexity. Since then, the DH and MSA theories have been the most popular nonempirical approaches used in the context of EoS modeling, due to their ease of implementation and low computational cost. Until now, there have been no detailed simulation benchmarks for the examination of these classic theories as perturbation terms that extend EoS models.

Several noteworthy studies have been focused on the use of the MSA theory to develop models for use in simulations of electrolyte solutions. Lamperski and Płuciennik^38^ proposed a model for electrolyte solutions in which the anions, cations, and solvent are represented as hard spheres (HSs) immersed in a dielectric continuum. Each of the ions was modeled with a single HS and a central point charge. In the case of the solvent, however, a given HS corresponds to a number of water molecules, or a molecular cluster. The authors assumed that the size of the HS representing a solvent cluster depends on the type of ions present in the system. Therefore, in the development of their model, they treated the diameter of the solvent HS as an adjustable parameter. They used their model to carry out both MSA calculations and inverse grand canonical Monte Carlo simulations for the mean ionic activity coefficient of aqueous NaCl and found that results from both the theory and the simulation were in good agreement with experimental data. In a separate study, Liu et al.^39^ analyzed the internal energies, pressures, and chemical potentials of model fluids of varying complexity (Stockmayer, Stockmayer and Lennard-Jones (LJ) mixtures, and ion–Stockmayer mixtures), comparing results from MSA theory with Monte Carlo simulation data. Their comparisons revealed that MSA calculations always overpredict the chemical potential and internal energy of dissolved ions.

The second contribution to the electrostatic terms comes from the ion–solvent interactions, and its consideration is essential to deliver accurate solvation properties.^5,9^ The Born theory of solvation^40^ is a primitive approach that can be used to model the effect of the solvation forces. Assuming the solvent to be a dielectric continuum characterized by a dielectric constant, the Helmholtz free-energy contribution that corresponds to the charging of ions in the solvent is obtained by integrating the Coulombic potential. In the calculation, the ions are considered to create cavities of fixed radius in the dielectric medium. The Born theory can be used to deliver accurate values of the experimental solvation energies of different ions when used with cavity radii which are slightly larger than the ionic radii.^41^ However, this approach to estimating experimental solvation energies is considered to be incomplete without explicitly considering the contributions that arise from the short-range repulsion and dispersion interactions.^42^

In several EoS studies,^10,11,43−50^ in which the solvent is modeled explicitly only in terms of its short-range repulsion, dispersion, and association forces, but without explicitly accounting for its polar nature, the ion–solvent long-range interactions have been treated using the Born expression. This simple strategy has been successfully incorporated in the cubic plus association (CPA)^43,44^ and several SAFT approaches.^10,11,45−50^ All these works reflect a consensus on the critical importance of incorporating an accurate description of the dielectric constant, as they demonstrated that good agreement with experiments can only be achieved using accurate empirical^9,11^ or semiempirical^51^ models for the relative permittivity of the solutions. However, although the importance of the dielectric constant is widely accepted, there is less agreement as to which other factors are the most important when developing EoS models for electrolyte solutions. For example, in EoS such as ePC-SAFT^8,52^ and the revised ePC-SAFT,^53^ the extended DH or MSA approaches are incorporated to treat the ionic interactions but the Born contribution is not considered. Although this approach leads to models that yield satisfactory results for several properties including the vapor pressure, mean ionic activity coefficient, and osmotic coefficient, it is not as effective when modeling the free energies of solvation, and liquid–liquid equilibria. By contrast, in explicit models, such as the electrolyte extension of PC-SAFT of Herzog et al.,^45^ and the SAFT-VR+DE of Zhao and co-workers,^54,55^ nonprimitive models for the electrostatic contribution have been used, leading naturally to the inclusion of the electrostatic ion–solvent interactions. Such methodologies yield excellent results, including the free energy of solvation. In a recent review, Kontogeorgis et al.^56^ provide a detailed overview of EoSs that employ the Born theory as a perturbation term to account for solvent–ion interactions.

The Born theory of solvation has been considered in several molecular-simulation studies,^57−60^ most of which were focused on improving or developing methodologies to estimate an effective Born cavity diameter of the ions modeled. For example, Babu and Lim^58^ used molecular-dynamics simulations to estimate the effective Born radius for ions in aqueous solutions. They simulated several strong aqueous solutions of alkyl halide (with TIP3P^61^ as the water model) and computed the Born cavity diameter as the mean between the experimentally measured ion diameter^62^ and the location of the first peak of the cation–oxygen and anion–hydrogen radial distribution functions. Lynden-Bell and Rasaiah^57^ developed a methodology in which the charge and the size of the ions are treated as dynamic parameters. This new approach allowed for the prediction of the entropy and enthalpy of solvation for several monovalent aqueous salts and improved the understanding of the asymmetries observed in such properties between anions and cations. Mongan et al.^59^ have compared several approaches to computing approximate solutions of the Poisson–Boltzmann equation. As a result, they obtained effective Born diameters, which were then used in computationally efficient molecular-dynamics simulations. The authors compared their own proposed approach with several others and found that it yields good results with data from explicit simulations of a variety of solvents. Most recently, Duignan and Zhao^60^ have shown that one can model the free energy of solvation of monovalent ions using Born theory calculations. To achieve this, they developed a symmetric water model to ensure symmetric solvent structure around the anions and the cations.

In a recent and very relevant article, Simonin^63^ explored the use of the Born theory of solvation to describe the ion–solvent interactions and proposed a modification using a scaling factor. The author showed that the Born term is too large when calculating properties that require rescaling using an infinite-dilution reference (e.g., the mean-ionic activity coefficient). Simonin also compared the Born term with the solution of the nonprimitive MSA for ion–dipole interactions (ID-MSA), showing that ID-MSA can exhibit negative trends in the concentration dependence of the rescaled chemical potential, while the Born term is always positive. This difference in the ion–dipole contribution of the rescaled chemical potential is an intriguing result, although, to the best of our knowledge, there is no evidence of its validity from simulation.

As with the theoretical calculations, computer simulations of electrolyte solutions are also hindered by the long-range nature of the Coulombic potential. Specifically, free-energy calculations, if not appropriately conducted, may become either highly inefficient or even yield incorrect results.^64^ The standard approach to performing free-energy calculations is through perturbations of the potential energy.^65^ However, in computer simulations, speed and robustness play a key role in determining the usefulness of a method. The need for fast and robust simulation methods for these systems is still relevant today, and this remains an active area of research.^31,64,66−69^

The simplest approach to measure the chemical potential (commonly used for simple liquids with nonpolar components) is the Widom ghost-particle insertion method,^70^ in which the molecules or particles of interest are inserted randomly in an equilibrated trajectory of *N* particles to sample the resulting change in potential energy. Unfortunately, when used to measure the chemical potential of charged species, Widom’s method is often slow to converge, and more importantly, it can even lead to inaccurate calculations.^71,72^ The approach does not work for systems characterized by long-range Coulombic interactions because most high-probability low-energy conformations of the *N*-particle system are too energetically unfavorable to appear in the (*N* + 1)-particle system. This discrepancy of probability makes averaging the difference between the two states extremely challenging.

Therefore, in studies involving aqueous electrolytes, more sophisticated approaches that introduce some bias to guide the sampling during the simulation are usually recommended. Examples of such methods are Bennet’s acceptance ratio,^73,74^ metadynamics,^75^ and expanded-ensemble (EE)^31,67,72,76^ simulations. The expanded ensemble transition matrix (EETM) method^71,72,76^ is based on an EE simulation where the Coulombic potential of the test particles is gradually switched on with weighted Monte Carlo moves. The weights introduce a bias in order to ensure that all intermediate sub-ensembles are sampled appropriately. These weights are generally unknown at the beginning of the simulation, but they can be estimated using a Wang–Landau (WL) algorithm.^77^ After producing a relatively accurate initial guess, the weights can be calculated directly by averaging the transition probabilities between the different sub-ensembles of the EE simulation.

In the current study, we benchmark primitive models commonly used to account for the Helmholtz free energy of electrolyte solutions. We choose to conduct our study by comparing the electrostatic contributions of the chemical potential of aqueous NaCl. The chemical potential is a first-derivative property and can be interpreted as the free energy per salt molecule. First, a model for an aqueous solution of NaCl that incorporates coarse-grained (CG) water beads^78^ is simulated. In this system, water is modeled simply as a LJ sphere without charges or dipole and, as a result, is subject only to short-range ion–solvent interactions. Thus, the free energy contribution arising from ion–ion interactions is isolated and can be compared to the predictions of the MSA^14^ and DH^13^ theories. We then turn to the aqueous NaCl model of Benavides et al.,^23^ where water is treated with the TIP4P/2005^79^ model (i.e., a polar water). The chemical potential obtained from simulations in this case can be used to benchmark the theoretical predictions generated by summing the Born and MSA free-energy terms. We conclude by presenting a complementary discussion on the fluid-structure of the CG and the TIP4P/2005 system obtaining their radial distribution functions and local orientation histograms around the ions.

The remainder of the article is set out as follows: In section 2 we present a brief overview of the primitive theories (DH, MSA, Born) examined in the current work. In section 3, we discuss the simulation methodology to conduct EE simulations for the electrostatic contribution to the chemical potential of the salt, and provide details of the two force fields used in the current study for the aqueous solutions of NaCl. Results are presented in section 4: In section 4.1 we focus on benchmarking the ionic free-energy contributions of the DH and MSA theories. In this context, the CG water force field is used, as there are no electrostatic interactions between the water and the ions in this model. In section 4.2 we turn to the investigation of the accuracy of the Born term. The TIP4P/2005 water model is used in order to introduce electrostatic interactions between the ions and the water molecules. Although the contribution from ion–ion interactions are not zero, these are shown to be negligible when compared to the Born contribution. An alternative approach is also investigated in which we attempt to model the chemical potential of the salt only using MSA. In section 4.3, the fluid structure of the two model systems is presented. We show the radial distribution functions, and (for the case of the TIP4P/2005 water) the local orientation of water around the ions. Finally, conclusions are presented in section 5.

## 2 Theoretical Approaches for Electrolyte Solutions

In this section, we briefly review the theories tested in our current work: the DH theory,^13^ the unrestricted MSA in the primitive model,^14^ and the Born theory of solvation.^40^ As discussed in the introduction, these approaches are selected because they are commonly used choices of primitive model used to incorporate the thermodynamics of electrolytes in EoS models.

### 2.1 Debye–Hückel Model

The DH^13^ free-energy expression is derived by solving Poisson’s equation assuming an electroneutral mixture of fully dissociated spherical ions dispersed in a continuum medium of given dielectric constant. An expression for the charge density around each ion^3^ is obtained assuming that the radial distribution function (RDF) of the ions follows a Boltzmann distribution based on the pair interaction energy, given by Coulomb’s law:
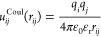
1where *r*_*ij*_ is the center–center distance between ions *i* and *j*, ε_0_ is the vacuum permittivity, ε_r_ is the dielectric constant, and *q*_*i*_ and *q*_*j*_ are the respective ion charges. This approximation for the RDF is exact in the limit of vanishing ion density, but the zero-density limit naturally neglects any packing effects. In the commonly used DH limiting law, the ions are considered as point charges,^80^ while a more general (commonly referred to as extended or full) description of the system can be obtained by modeling the ions as charged hard spheres. The full DH expression for the Helmholtz free energy contribution is given as^13,19^
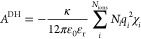
2where *N*_ions_ is the number of ion types present in the system and *N*_*i*_ is the number of ions of type *i*. κ denotes the inverse screening length,^13^ which is a measure of how far the electrostatic effect of ion *i* persists; this is given by
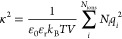
3where *k*_B_ is the Boltzmann constant, *T* is the absolute temperature, and *V* is the volume. In addition, χ_*i*_ in eq 2 is the so-called auxiliary function:
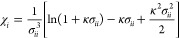
4where σ_*ii*_ is the hard-sphere diameter of ion *i*.

### 2.2 Mean Spherical Approximation (MSA)

Blum^14^ was the first to present a solution of the Ornstein–Zernike^81^ integral equation using the MSA closure for an electroneutral mixture of charged hard-spheres of arbitrary diameter (unrestricted), immersed in a dielectric continuum (primitive model). The solution leads to a set of equations that can be solved numerically for self-consistency.

Following the MSA approach for ions solvated in a continuous dielectric medium, the expression for the Helmholtz free energy is given as
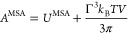
5where *U*^MSA^ is the MSA internal energy and Γ is the screening length of the electrostatic forces. The MSA internal energy is given by
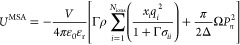
6where the sum is over all ion types, as before, and Δ is related to the packing fraction of the ions and is hence a function of the diameter σ_*ii*_ of the ions:
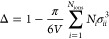
7The functions *P*_*n*_ and Ω are auxiliary quantities, given by
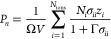
8
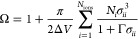
9The screening length Γ is a function of the dielectric constant and the effective charge *Q*_*i*_:
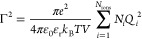
10and
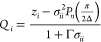
11where *e* is the elementary charge and *z*_*i*_ is the valency of ion *i*. The screening length Γ is obtained through an iterative procedure that ensures self-consistency between eqs 5 and 10. Here, we use the Newton–Raphson method, with the DH inverse screening length κ as an initial guess Γ_0_:
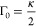
12

### 2.3 Born Theory of Solvation

The Born theory of solvation^40^ is a primitive approach for the description of the free energy arising from ion–solvent interactions. The solvent is treated as a continuous dielectric medium and the free energy of inserting an ion in the solvent is calculated as the work needed to open a cavity (the so-called Born cavity) in the medium. Under the approximation that each new ion inserted does not affect the properties of the medium around the other ions, an equation can be obtained to treat electrolyte solutions involving multiple ions. The Born free-energy term is thus given as
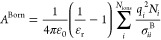
13where σ_*ii*_^B^ is the diameter of the Born cavity associated with ion *i*. Equation 13 can also be interpreted as the free energy required to bring an ion into the solvent of interest from vacuum.^10^

It is relevant to note that in the Born model, the charge of the ion is assumed to be distributed on the surface of the Born cavity. Given the expression derived by Born, smaller ions interact more strongly with their environment, corresponding to an increase in the Coulombic attraction at smaller *r*_*ij*_ (see eq 1). A further important consideration is that the Born cavity is assumed to be rigid in the theory. As described by eq 13, beyond the surface of the Born cavity, the Coulombic force is scaled by the macroscopic dielectric constant of the solution (ε_r_), and as a result, the impact of packing effects on the local structure of the solvation shell around the ions is implicitly approximated as negligible.

### 2.4 Electrostatic Chemical Potential

The chemical potential μ_*i*_ of a given compound *i* can be obtained from the Helmholtz free energy following a standard thermodynamic relation:
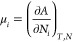
14and the electrostatic contribution to the total chemical potential is given by the sum of the ionic and Born terms,
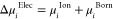
15where μ_*i*_^Ion^ refers to the ion–ion contribution, treated at either the DH or MSA level in our current work, and μ_*i*_^Born^ refers to the Born contribution to the chemical potential. In the case of a monovalent salt *S*, the electrostatic contribution is given as
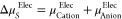
16assuming complete dissociation of the cation and anion, assumed in both the MSA and DH models.

We also note that the chemical potential commonly appears in a rescaled form using an infinite dilution state as reference:
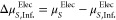
17where μ_*S*,Inf_^Elec^ is the chemical potential at the reference state of infinite dilution. In this reference state, only a single ion, either a cation or an anion, is solvated in an infinitely large system.

## 3 Simulation Methods

### 3.1 Expanded-Ensemble Simulations

The configurational integral of an *N*-particle system in the canonical ensemble (constant number of particles (*N*), volume (*V*), and temperature (*T*)) is given by
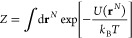
18where *U* is the total potential energy of the system and **r**^*N*^ are the generalized position vectors of all the particles. Let us consider two states for the (*N*, *V*, *T*) system such that they are characterized by two values of a coupling parameter λ:  and , where β = 1/(*k*_B_*T*). The difference in the Helmholtz free energy between these two states at the same temperature can be written as
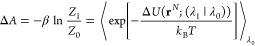
19where  denotes an ensemble average in the reference state λ_0_. The system of interest is simulated at state (*N*, *V*, *T*; λ_0_), and its state is perturbed to (*N*, *V*, *T*; λ_1_) to compute the energy change Δ*U*(**r**^*N*^; (λ_1_|λ_0_)). Once this computation is done, the system state is reverted to the (*N*, *V*, *T*; λ_0_) state, and the simulation is continued. A large number of states are ensemble-averaged to obtain the free-energy difference. This procedure yields accurate free-energy estimates when the corresponding microscopic states of the target (λ_1_) can be visited reasonably frequently from the reference state (λ_0_). In other words, the procedure results in reliable estimates of the free energy if the free-energy barrier is lower than the thermal fluctuations; otherwise, a more robust procedure is needed.

In order to carry out the chemical-potential calculations required to benchmark the theoretical approaches discussed earlier, we use the EE transition matrix (EETM) method.^31,67,76^ To calculate the change in free energy caused by the Coulomb interaction of an ion pair, we focus on the free-energy path of charging and discharging a randomly selected pair of ions. The ion charges are scaled using a linear path between the state with test particles uncharged (λ = 0) and the corresponding charged state (λ = 1). In our methodology, λ assumes discrete values, with each value defining a separate *NVT* sub-ensemble, while in other methods such as in metadynamics^75^ the collective variable is continuous. The configurational integral for each sub-ensemble *i* is simply:
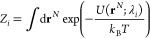
20

We can now define an expanded ensemble (EE) of the sub-ensembles, with the configurational integral of this new EE defined as
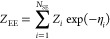
21where an (at this point) arbitrary weight η_*i*_ is assigned to each sub-ensemble, and *N*_SE_ denotes the number of sub-ensembles that comprise the expanded ensemble. The transition between sub-ensembles is implemented as Monte Carlo (MC) moves that are accepted or rejected using the standard Boltzmann criterion as implied by eq 21. Each individual sub-ensemble may be simulated with molecular-dynamics (MD) or MC simulations, as it is an independent *NVT* ensemble.

In order to explore the relationship between the Helmholtz free energy and the weights η_*i*_, we proceed with the assumption that the weights have been appropriately chosen to force equiprobable sampling of the phase space of the new expanded ensemble. This equiprobable sampling implies that the biased macroscopic probabilities (*P*) of visiting each sub-ensemble are equal:
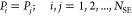
22where the subscripts *i* and *j* denote the sub-ensemble. It immediately follows that the configurational integrals of any two sub-ensembles need to be equal as well. This leads to an expression for the difference in Helmholtz free energy between sub-ensembles:
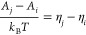
23

One can interpret from eq 23 that the EE simulation will enable one to equiprobably sample the sub-ensembles, only if the weights η_*i*_ correspond to the free-energy differences between the sub-ensembles. Therefore, the task of computing the free energy is equivalent to finding a set of weights that flatten the histogram of visited states. In practice, only moves between adjacent sub-ensembles are considered. To compute the total free-energy difference, one must then sum all of the intermediate free-energy differences at the end of the simulation.

### 3.2 Transition Matrix Monte Carlo

In order to compute efficiently the desired Helmholtz free-energy difference between an uncharged and a fully charged pair of ions (one cation and one anion in the case of monovalent salts), the transition matrix Monte Carlo (TMMC) method is employed.^31,67,76^ TMMC is based on sampling directly the transition probabilities between different sub-ensembles of the EE. The microscopic unbiased transition probabilities (ϖ_*i*→*i*+1_) are averaged in order to calculate the corresponding unbiased macroscopic transition probabilities (*Π*_*i*→*i*+1_). In the context of our study, only transitions between adjacent sub-ensembles are considered.

Consider the detailed balance of the macroscopic probabilities:
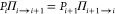
24We may rewrite this as
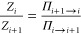
25and introduce the free-energy difference between the two unbiased isothermal states (Δ*A*_*i*→*i*+1_) as the ratio of the two configurational integrals (cf. eq 19):
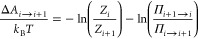
26The macroscopic probabilities are averaged using a collection matrix *C* such that whenever a sub-ensemble change is attempted, the microscopic unbiased transition probabilities are collected according to
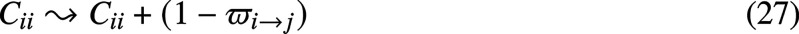
27
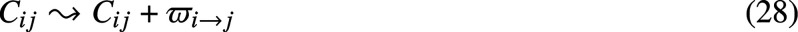
28where row *i* denotes the origin sub-ensemble, and column *j* denotes the target sub-ensemble. In addition, the curly right arrow denotes an update of the value of the left-hand side to the value on the right-hand side.

The TMMC sub-ensemble step is presented in Algorithm 1. The collection matrix needs to be updated every time a new move is attempted within the MC simulation, regardless of whether the move is accepted or not, as even the rejected states contribute to the ensemble average. Furthermore, when a sub-ensemble change is attempted, not only the element of the collection matrix associated with the transition but also its complementary element are updated. Specifically, the complementary probability of leaving a sub-ensemble is the probability of staying, as their sum should always be equal to one. The complementary probabilities should also be stored in the collection matrix, as their inclusion in the averaging speeds up the calculation significantly. In EE simulations, a range of appropriate values is chosen for the collective variable λ. In our current work λ, which varies from 0 to 1, is used to scale the ionic charges according to λ*q*_*i*_. Any move where one attempts to change the collective variable value to a value that is out of the chosen range is simply rejected and the current ensemble is penalized.

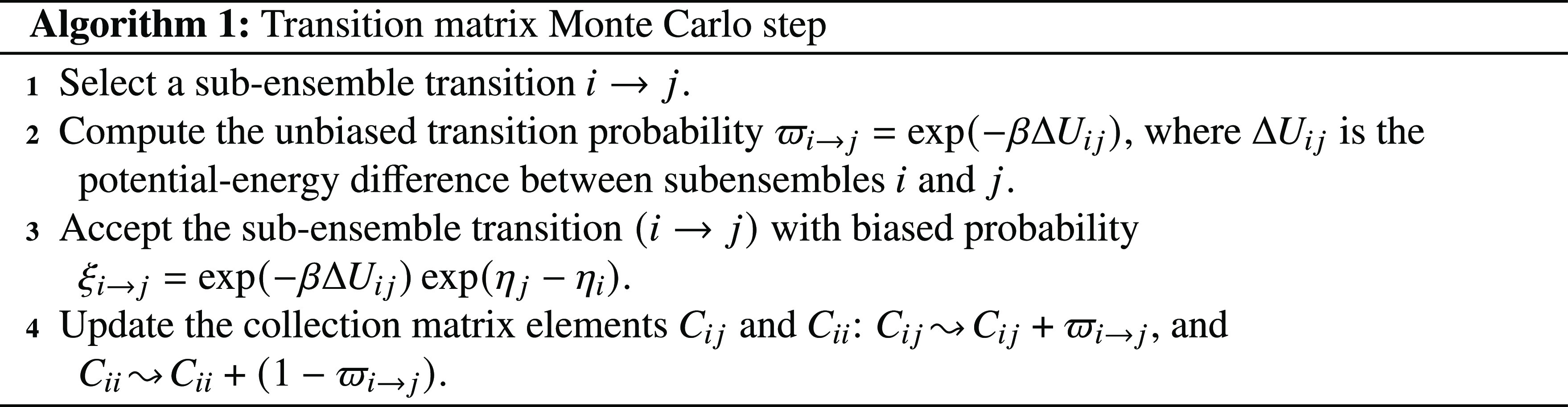



Finally, the macroscopic probabilities may be calculated from the collection matrix by
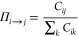
29where the sum over *k* includes all the sub-ensembles from *i* – 1 to *i* + 1.

Following this methodology the electrostatic chemical potential is computed from the averaged macroscopic transition probabilities of TMMC, as they are directly related to the free-energy landscape of the collective variable λ:
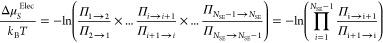
30where Δμ_*S*_^Elec^ is the electrostatic contribution to the chemical potential (Helmholtz free energy) of a pair of ions, which represents a fully dissociated salt molecule. This accounts only for the ion–ion and ion–solvent electrostatic interactions, as the reference state in our EE simulation is the state where the charges of the test ion pair are both zero, but the ions are still present in the system as LJ spheres.

### 3.3 Wang–Landau Sampling

At the start of the EE simulation the weights of the EE simulation are unknown. In order to provide an initial estimate of the chemical potential, a Wang–Landau (WL)^77^ scheme is employed, in which all the weights are initialized to zero. After every MC move that results in a visit to a given sub-ensemble *i*, the weight η_*i*_ is updated with a small penalty γ, so that the probability of revisiting sub-ensemble *i* is reduced:


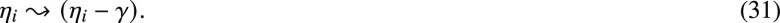
31

All of our calculations start with an initial large value of γ in order to force the simulation to quickly explore all of the sub-ensembles. The WL scheme is then kept running until the slope of the histogram of the number of visits to each sub-ensemble is less than a prescribed value, *s*_c_. In practice, *s*_c_ is commonly chosen to be close to 1 to enforce a uniform histogram. Once this threshold is achieved a new scaled γ is used. In our current work, the factor used to scale γ between different WL iterations is denoted by α:


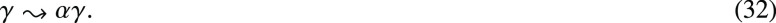
32

As what could be considered a satisfying accuracy for the WL initial guess depends on the system of study, the minimum value of γ is reported as part of the simulations details in section 3.4 alongside the force-field parameters and other system-specific information for the simulation. An algorithmic representation of a Wang–Landau transition is presented in Algorithm 2.

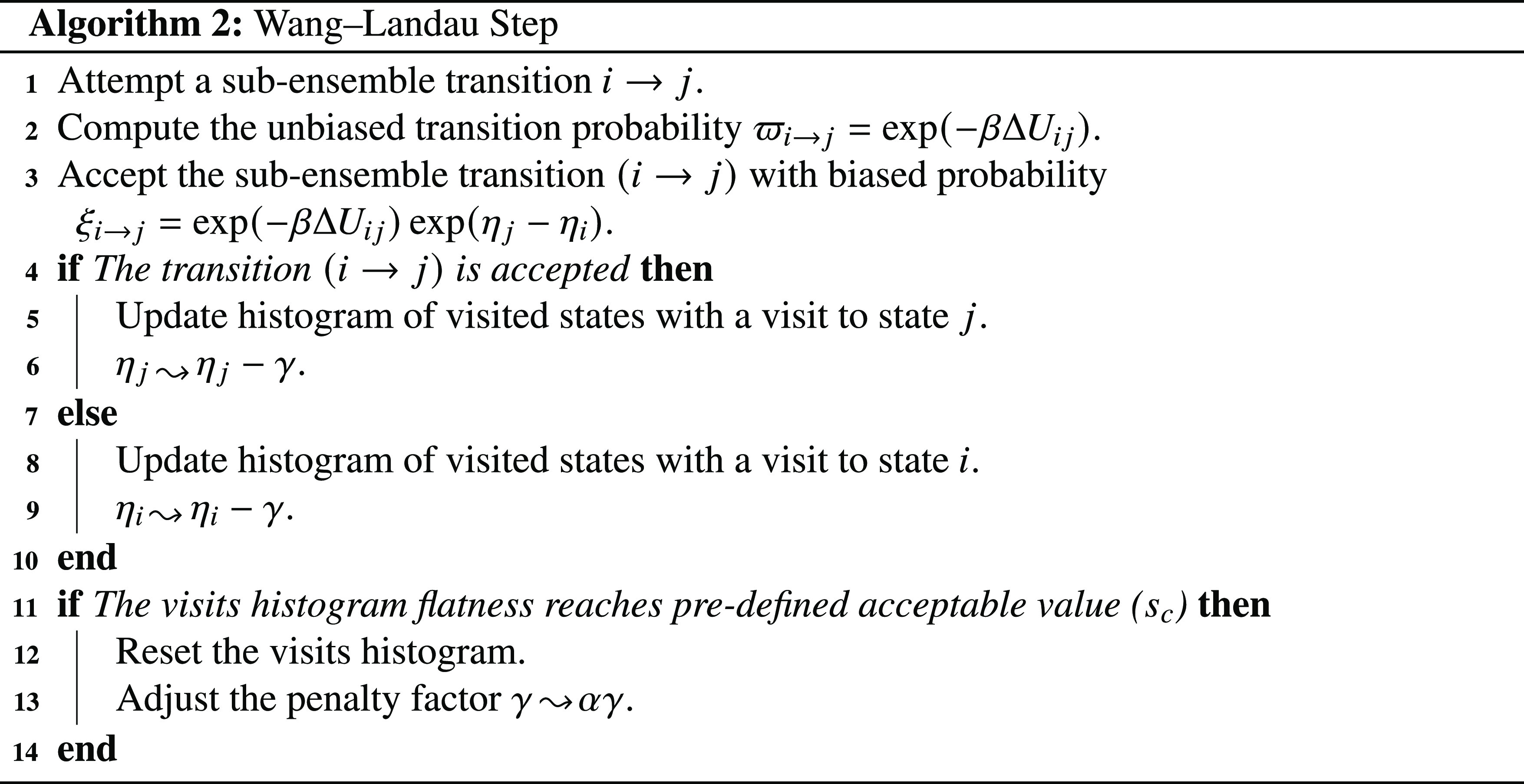



### 3.4 Molecular Models and Simulation Details

We study aqueous solutions of NaCl, using two different models. The first model is taken from the work of Andreev et al.,^78^ in which water, Na^+^, and Cl^–^ are modeled as spherical Lennard-Jones particles. Water is treated as nonpolar, i.e., the model does not include explicit polarity or charges. The ions are modeled as charged LJ spheres of identical size to the solvent and are distinguished only by having different unlike LJ interaction energies with the water particles. We refer to this model as coarse-grained LJ (CG/LJ) for convenience. Our second model corresponds to the JC/TIP4P/2005 of Benavides et al.,^22^ which incorporates the TIP4P/2005^79^ water model, i.e., a polar model with charges on the hydrogen and oxygen (off-center) atoms included, with the ions treated as LJ spheres with central point charges.

The LJ potential *u*_*ij*_^LJ^ between particles *i* and *j* is given by
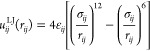
33where *r*_*ij*_ is the center–center distance, σ_*ij*_ is the separation at which the potential is zero (the particle diameter), and ε_*ij*_ is the depth of the potential well. The point charges of the ions and the partial charges of the TIP4P/2005 water are assigned electric charges that interact through the Coulomb potential (cf. eq 1).

The use of the two models selected allows one to isolate each of the electrostatic contributions to the chemical potential. In the CG/LJ model of Andreev et al.,^78^ a (coarse-grained) water particle with no explicit polarity is used, such that the entire electrostatic contribution to the chemical potential of the ions comes only from the ion–ion Coulomb interactions. This allows an assessment of the accuracy of the MSA and DH theories for the calculation of the ion–ion contribution. By contrast, particularly for the low-concentration electrolyte solutions of interest here, the chemical potential of the ions in the JC/TIP4P/2005 model of Benavides et al.^22^ is dominated by the ion–solvent interactions, because the solvent (water) is the main component. This model allows us to assess the performance of the Born term in comparison with simulation. Unless otherwise stated, all of the calculations in our current work involve charge perturbations of a randomly chosen test ion pair that represents a fully dissociated salt molecule. Hence, hereafter, the chemical potential of an ion pair is referred to as the chemical potential of the salt μ_*S*_.

In section 4.2, we report the dielectric-constant calculations for the JC/TIP4P/2005^22^ model over a range of salt concentrations and temperatures. The calculations are conducted by averaging the fluctuations of ensemble averages of the total dipole moment **M** (polarization), given by
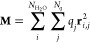
34where  is the number of water molecules in the system, *N*_*q*_ = 4 is the number of charged sites on each TIP4P/2005 water model, and *q*_*j*_ and **r**_*i*,*j*_ are respectively the charge and Cartesian coordinates of each charged site *j* of the water molecule *i*. The dielectric constant is obtained from the fluctuations of **M** as^82^
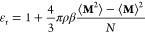
35where the angular brackets denote an *NVT* ensemble average. In practice, many configurations of the system are postprocessed and both the total dipole moment, **M**, and its square, **M**^**2**^, are averaged. Subsequently, the dielectric constant is computed using eq 35. An indication of sufficient averaging is that the result for the average of the total dipole moment ⟨**M**⟩ is expected to be zero, as for all of the thermodynamic states considered as there is no permanent orientation of the water molecules, i.e., the aqueous phase in not polarized. This approach is convenient as it allows the assignment of a “tin foil” conductor as the surrounding medium. In addition, it has been shown that for sufficiently large simulation boxes the error in the calculation becomes negligible.

#### 3.4.1 Coarse-Grained Lennard-Jones Model for Aqueous NaCl

Andreev et al.^78^ presented a coarse-grained model for several types of ions in water. In their model, both the ions and water are considered to be identically sized spherical LJ beads. In addition to their designated charge, the ions are distinguished from one another by a single parameter only, corresponding to their cross-dispersion energy with water, which was adjusted based on free energy of solvation data. The water particle is not assigned any charge; it is represented simply as a neutral spherical LJ site. The LJ parameters for this model are given in Table 1 in dimensionless units defined as
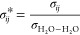
36Although as all of the spheres are of the same size, we drop the subscripts here and use only σ* for the reduced diameter; the well depth is scaled as
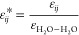
37

**Table 1:** Force-Field Parameters for the CG/LJ Model of Andreev et al.^78^^,^^a^

molecule	molecule	σ*	ε_ij_^*^
H_2_O	H_2_O	1.00	1.00
Na^+^	Na^+^	1.00	1.00
Cl^–^	Cl^–^	1.00	1.00
H_2_O	Na^+^	1.00	1.25
H_2_O	Cl^–^	1.00	1.00
Na^+^	Cl^–^	1.00	1.00

a The model is presented in dimensionless units of  and . The same set of parameters is used when performing calculations with the MSA and DH theories.

The ion–ion Coulombic interactions of the model are given in dimensionless units by fixing the Bjerrum length, which is the separation distance between two ions at which Coulombic interactions are balanced by the thermal energy of the system. In our current work we use dimensionless units, and the Bjerrum length is reduced using the diameter of water. It is thus given as
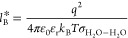
38The same ionic strength can be achieved by adjusting either the value of the ion charges or the dielectric constant, at a given temperature. In practice, we simulate a system at a fixed dielectric constant equal to 1 (vacuum permittivity), and adjust the charges of the anions and cations to match the desired Bjerrum length at each given temperature.

In all of the simulations involving the CG/LJ model there are *N* = 2000 particles in a cubic periodic simulation box of edge length equal to 13.43σ*, which is equivalent to . In addition, all simulations are conducted at . Andreev et al.^78^ proposed a mapping between the LJ fluid and the aqueous solutions of NaCl that reproduces the critical temperature and pressure of water. In this mapping, the chosen simulated state corresponds to a solution at 420 K and 1 atm. (In the original publication of Andreev et al.,^78^ there is a typographical error: The temperature is erroneously reported to be 320 K.)

In order to generate initial configurations for our simulations, we insert the desired atoms in a crystal, and before running any MD simulation, we perform random swaps to guarantee there are no ion clusters that might increase unnecessarily the equilibration time. Subsequently, the crystal is melted to an equilibrated state using an *NVT* simulation of 1 ns (using a time step of 2 fs). *NVT* simulations of 20 ns are then performed, starting with the equilibrium configurations, to compute the RDFs of the mixture.

The EE simulations for the calculation of the chemical potential, which are also started from the equilibrated configurations, are carried out for 4 ns, with a MC sub-ensemble change attempted every 40 fs. As mentioned in section 3.1, in the initial stage of the EE simulations, good estimates of the free-energy profile are obtained using the WL^77^ approach. The flatness acceptance parameter is set to *s*_c_ = 0.95, and the scaling factor is set to α = 0.5. We start the simulation with γ = 1. Once γ reaches a value of less than 10^–4^, the WL approach is terminated, and the guesses from the TMMC approach are used to refine the free-energy profile. All of the MD simulations are conducted using the Large-scale Atomic/Molecular Massively Parallel Simulator (LAMMPS).^83^

#### 3.4.2 JC/TIP4P/2005 Model for Aqueous NaCl

In the second model for the aqueous NaCl solution, the TIP4P/2005 model^79^ is used to represent water, and the JC/TIP4P/2005^22^ model is used for the ions and ion–water interactions. This force field was parametrized to reproduce the experimental solubility of NaCl in water at 298 K.^22^ The TIP4P/2005 water model is a four-site model with a LJ site placed on the oxygen atom, and three partial charges placed, one on each hydrogen, and one on an off-center virtual site (M). The values for the parameters in this force field are given in Table 2.

**Table 2:** Force-Field Parameters of the JC/TIP4P/2005 Model.^22^^,^^a^

atom	atom	*q* (*e*^–^)	σ_*ij*_ (nm)	ε_*ij*_ (kcal/mol)
O	O		0.31589	0.18520
M	M	–1.1128		
H	H	+0.5564		
Na^+^	Na^+^	+1.0000	0.21600	0.35262
Cl^–^	Cl^–^	–1.0000	0.48300	0.01279
O	Na^+^		0.26590	0.25555
O	Cl^–^		0.39940	0.04866
Na^+^	Cl^–^		0.34950	0.06714

a There are four interaction sites on the TIP4P/2005 model of water. Three of them are placed at the center of the oxygen and hydrogen atoms, respectively. The final site, often called the M site, is co-planar with the O and H sites and is located at the bisector of the H–O–H angle. The O–H distance is fixed to 0.09572 nm, and the H–O–H angle is fixed to 104.52°. The same set of parameters is used when performing calculations with the MSA and DH theories.

Each system is initially equilibrated at the desired temperature and pressure for 2 ns through an *NpT* simulation with a time step of 2 fs, allowing the system to relax to its equilibrium density. The equilibrated system is then used as an initial state for an *NVT* simulation of 10 ns. This 10 ns trajectory is used to compute the reported RDFs, the orientational histograms, and the dielectric-constant data. To conduct the constant-temperature or constant-pressure MD simulations, the Nosé–Hoover thermostat and barostat are used, as described in the LAMMPS^83^ manual.

The final frame of the equilibration simulation is also used as an initial configuration to perform the EE simulations for the chemical potential of the salt. In these runs, we simulate the system for 2 ns, with breaks for an MC sub-ensemble change attempt every 50 fs. The general methodology is that described in section 3.1. For the WL step, the flatness acceptance parameter is set to 0.8, and the scaling factor is set to 0.1. During the simulation, once the scaling factor reaches a value of less than 10^–2^, the WL approach is terminated, and the guesses from the TMMC approach are used to refine the free-energy profile.

## 4 Results and Discussion

This section is divided into three parts. First, in section 4.1 we compare the MSA and DH contributions to the chemical potential of NaCl salt with simulation data generated using the CG/LJ model.^78^ Then in section 4.2, we benchmark the Born theory of solvation by comparing theoretical predictions for the chemical potential of the salt with simulations generated using the JC/TIP4P/2005 model.^22^ Finally, in section 4.3 the molecular structure of the two models considered is investigated by computing RDFs and local orientational histograms. Here, particular emphasis is placed on investigating the relationship between the Born radius and the computed RDFs and orientational histograms.

### 4.1 Ion Term

The MSA^14^ and DH^13^ theories are used to compare the values of the chemical potential of ion pairs (one anion and one cation) with simulation data computed using expanded ensemble simulations.

For this comparison, the CG/LJ force field is chosen, as the water molecules are represented as simple uncharged LJ spheres in this model, a choice that does not consider the electrostatic forces between the ions and the solvent. Omitting the ion–solvent electrostatic interactions means that the Born free energy is zero, and the focus is solely on comparing the contributions arising from ion–ion interactions. Both the MSA and DH theories require the diameter of the ions to be defined; these are taken directly from the ion diameters as defined in the CG/LJ model (cf. Table 1). In addition, both the DH and MSA approach require a specification of the ion charges and the dielectric constant. In our current work, these quantities are implicitly set using the Bjerrum length, given by eq 38. When proposing the CG/LJ model, Andreev et al.^78^ set the Bjerrum length as *l*_B_^*^ = 1.85. It is important to note at this point that, as the Bjerrum length does not depend on ion concentration, carrying out calculations at a fixed *l*_B_ is equivalent to neglecting any dependence of the dielectric constant on the ionic concentration.

In our work, we expand the ionic strengths considered by conducting simulations not only for *l*_B_^*^ = 1.85 but also for an extended range that covers Bjerrum length values up to *l*_B_^*^ = 10. This upper limit is guided by the ranges covered in previous EoS and molecular-simulation studies reported in the literature; a number of indicative studies are presented in Table 3. As can be seen from the table, in modeling electrolyte solutions the SPC/E^84^ and the TIP4P/2005^79^ force fields lead to the underprediction of the experimental dielectric constant of water of 78.3 at 298 K.^22−24^ In EoS studies, no systematic deviation is observed because the dielectric-constant models used are adjusted to experimental data.^9,11,51^ For the simulation studies listed in Table 3, the Bjerrum lengths calculated using eq 38 fall in the range of 3–10 σ_Na–Cl_, where σ_Na–Cl_ is the contact distance between the sodium and chlorine ions. Experimental measurements and EoS models fall within the range of 2–3 σ_Na–Cl_. For all of the models in Table 3, the range of interest of the Coulomb interaction strength does not exceed *l*_B_ ≈ 10σ_Na–Cl_, where the σ_Na–Cl_ diameter is the one used in the corresponding reference as given in the table.

**Table 3:** Bjerrum Length (*l*_B_) and Dielectric Constant (ε_r_) of Various EoS and Simulation Models for Aqueous NaCl at 298 K and 1 atm^a^

model	*m* (mol/kg)	ε_r_	*l*_B_/σ_Na–Cl_	ref
SAFT-VRE Mie	inf. dil.	78.78	2.56	(10)
	5.0	72.94	2.90	
eSAFT-VR	inf. dil.	78.31	2.72	(11)
	5.0	78.31	2.72	
JC/TIP4P/05	inf. dil.	55.00	2.92	(22)
	5.0	24.50	6.54	
RDVH/SPC/E	inf. dil.	69.00	2.58	(84)
	5.0	24.30	7.32	

a The values given for the Bjerrum length are calculations from our current work, and have been computed using dielectric-constant calculations and model parameters as reported in the corresponding cited publications. The σ_Na–Cl_ diameters are taken from each model, as presented in the corresponding reference.

At this point, to help set our simulations in context, it is useful to conduct the reverse calculation, to establish the dielectric constant corresponding to the CG/LJ force field that we are using for the simulation benchmarks. Andreev et al.^78^ proposed that the value of the Bjerrum length be set to 1.85 σ, but to calculate the dielectric constant from eq 38, the charge of ions and the diameter σ need to be defined in real units. If we consider *q*^2^ = 1*e*, where *e* is one unit of electron charge, and a diameter σ = 0.2785 nm (taken from the SAFT-VRE Mie model),^10^ then the dielectric constant is obtained as 77.2, a value that is in the same range as those of the EoS models considered in Table 3.

**Figure 1:**
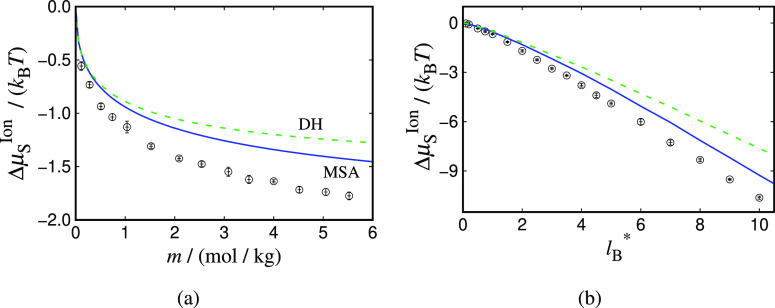
Coulomb contribution to the chemical potential as a function of: (a) the molality, *m*, of the solution for the CG/LJ system and *l*_B_^*^ = 1.85; and (b) the Bjerrum length, *l*_B_^*^, of the modified CG/LJ model (at *m* = 0.5 mol/kg). The symbols correspond to EETM simulations and the curves to theoretical calculations using the MSA (continuous, blue) and full DH (dashed, green) theories. The simulations are conducted at T* = 0.75 and ρ* = 0.8. The error bars correspond to the uncertainty of the simulation data.

In Figure 1 the theoretical and simulation data are presented for the CG/LJ model; plots of the chemical potential as a function of molality and as a function of Bjerrum length are given. The chemical potential of the salt is observed to decrease with an increase in the molality of the solution or the Bjerrum length. Both the MSA and DH theories successfully capture this qualitative behavior, but both fail to deliver accurate quantitative agreement. The quantitative agreement is better at low molalities or weak ionic strengths than at high molalities or strong ionic strengths, as both theories are formulated by assuming low-density approximations. As expected, the calculations from the MSA approach are in slightly better quantitative agreement than those from the DH approach.

In both theories, there is a limit at vanishing ion density, where the theories converge to the DH limiting law.^13^ As the concentration of the ions in solution is increased, the Coulomb contribution obtained using MSA is consistently closer to the simulation results. This finding demonstrates that, from a theoretical point of view, the MSA approach rests on a firmer physical footing than the DH approach. The trend of decreasing chemical potential observed with both approaches, as well as the simulations, is physically reasonable since, when ions are added to the solution, they interact through the strongly attractive Coulomb force. Moreover, it is interesting to note that the decrease of the chemical potential with salt concentration steadily becomes smaller as the molality of the solution is increased. The smallest value calculated is approximately −1.25 *k*_B_*T* for the MSA calculations at *m* = 6 mol/kg. This value of the chemical potential is of the same magnitude as the molecular kinetic energy fluctuations of the fluid, given by *k*_B_*T*. This observation is not surprising since the ions are dissociated only through molecular fluctuations, as the solvent is not explicitly polar and, therefore, cannot form oriented solvation shells. It is important to point out that when one uses a force field that incorporates a nonpolar model of water (not characterized by partial charges) a solvated salt must exhibit a chemical potential close to *k*_B_*T*, because uncharged particles can solvate ions only through dispersion interactions. To provide a point of reference, we note that the dispersion LJ interactions contribute approximately −5.2 *k*_B_*T* to the chemical potential (for the water and Cl atoms); this value is calculated using the SAFT-VR Mie^85^ EoS (we choose the point where *m* = 3 mol/kg from Figure 1), which is known to give excellent quantitative predictions when compared with simulation data.^85−88^

The MSA and DH theories have been compared against simulation data in previous studies. Our current results are consistent with the results of Valleau and co-workers.^32,33^ These authors found that both theories provide good qualitative agreement with simulation data for the osmotic coefficients (quantities related to the excess chemical potential) but that the agreement is not quantitative; this was attributed to the low-density approximation involved in their derivation. More recently, Maribo-Mogensen et al.^19^ have demonstrated that the difference between MSA and DH is not significant enough to be noticeable in the context of EoS thermodynamic modeling, as the additional error introduced from using the extended DH theory can be easily compensated by adjusting other parameters, such as the ion–water dispersion energy. This is consistent with the results of our simulations since although, as discussed in relation to Figure 1, the Δμ_*S*_^Ion^ values obtained using MSA are consistently more accurate, the deviations between these and the simulation data are more significant than the differences between the DH and MSA calculations of Δμ_*S*_^Ion^. It is worth noting that in several studies^34,37,54,89,90^ these theories have been modified using empirical or semiempirical approaches to compensate for this deviation from simulation. However, most of these studies were developed using osmotic or activity-coefficient data and not chemical-potential data.

### 4.2 Born Term

To assess the accuracy of the Born free-energy expression given in eq 13, the JC/TIP4P/2005^22^ model is used. As discussed in section 3.4.2, in the TIP4P/2005^79^ force field, water is modeled as a LJ sphere with partial charges to represent the H and O atoms, so the polarity of water is incorporated explicitly. As a result, besides the ion–ion interactions, an electrostatic contribution arises from the interaction between the partial charges on the water molecules and the ions. We carry out EE simulations, generating simulation data for the chemical potential of ion pairs (Δμ^Ion^ + Δμ^Born^) in TIP4P/2005 aqueous solutions of varying concentration and temperature. These are compared with data obtained from theoretical calculations using the Born and MSA theories.

We consider the Born and ion terms together as it is impossible to decouple these two contributions in a model in which water is charged. Therefore, in the context of our current work, the Born theory of solvation can be assessed only in combination with a model for the ion–ion interactions, unless we limit the discussion to ions at infinite dilution (where there is no ion–ion contribution). The ion diameters used to perform the MSA calculations are taken directly from the JC/TIP4P/2005^22^ force field (Table 2). In contrast to the CG/LJ force field (Table 1), each ion is characterized by a different diameter and contributes asymmetrically to the ion–ion chemical potential. In the calculation of the Born chemical-potential contribution, the diameter of the Born cavity also needs to be characterized. This so-called Born diameter is usually either related to the ionic diameter^41^ or adjusted^10,11^ using experimental data of the free energy of solvation. Later in this section, we will return to the challenge of choosing an appropriate value for the Born diameter. However, before proceeding, we need to fully define the inputs of the theoretical calculations by determining the dielectric constant of the fluid in the studied thermodynamic states.

**Figure 2:**
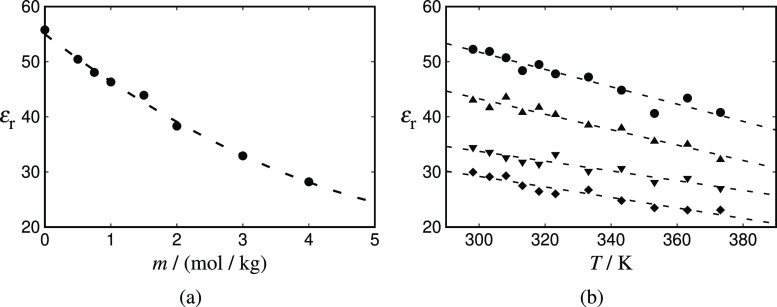
Static dielectric constant of aqueous NaCl as a function of: (a) salt molality, at *T* = 298 K; and (b) temperature for molality of *m* = 0.5 mol/kg (circles), *m* = 1.5 mol/kg (up triangles), *m* = 3.0 mol/kg (down triangles), and *m* = 4.0 mol/kg (diamonds). The symbols represent *NVT* simulation data for the JC/TIP4P/2005^22^ model. The simulations are carried out at fixed *V* averaged from separate *NpT* runs at *p* = 1 atm. The dashed curve in (a) is a quadratic correlation of the simulation data; those in (b) are linear correlations. The corresponding expressions are provided in the Supporting Information.

It is evident by examining eq 13 that the dielectric constant (ε_r_) of each state is needed as an input. In the current work, values of the dielectric constant at different molality and temperature are obtained carrying out *NVT* simulations (cf. section 3.4.2) for the JC/TIP4P/2005 model; the data are presented in Figure 2. As expected, the dielectric constant is seen to decrease with increasing molality, since the orientational ordering of the water molecules (caused by the presence of the ions) reduces the dielectric response of the solvent, a phenomenon referred to as dielectric saturation.^91,92^ Furthermore, an increase in temperature also leads to the decrease of the dielectric constant, although as is apparent from Figure 2, the decrease associated with increasing temperature is less pronounced than the change seen with increasing molality for the ranges considered here. The decrease in the dielectric constant of the solution with increasing temperature is due to the higher average kinetic energy, which hinders the water molecules from forming (orientationally ordered) solvation shells around the ions. The simulation data are correlated to linear or quadratic polynomials to reduce statistical noise and are used to generate values to be used in the corresponding theoretical calculations with the ionic and Born expressions (the correlations are provided in the Supporting Information).

**Figure 3:**
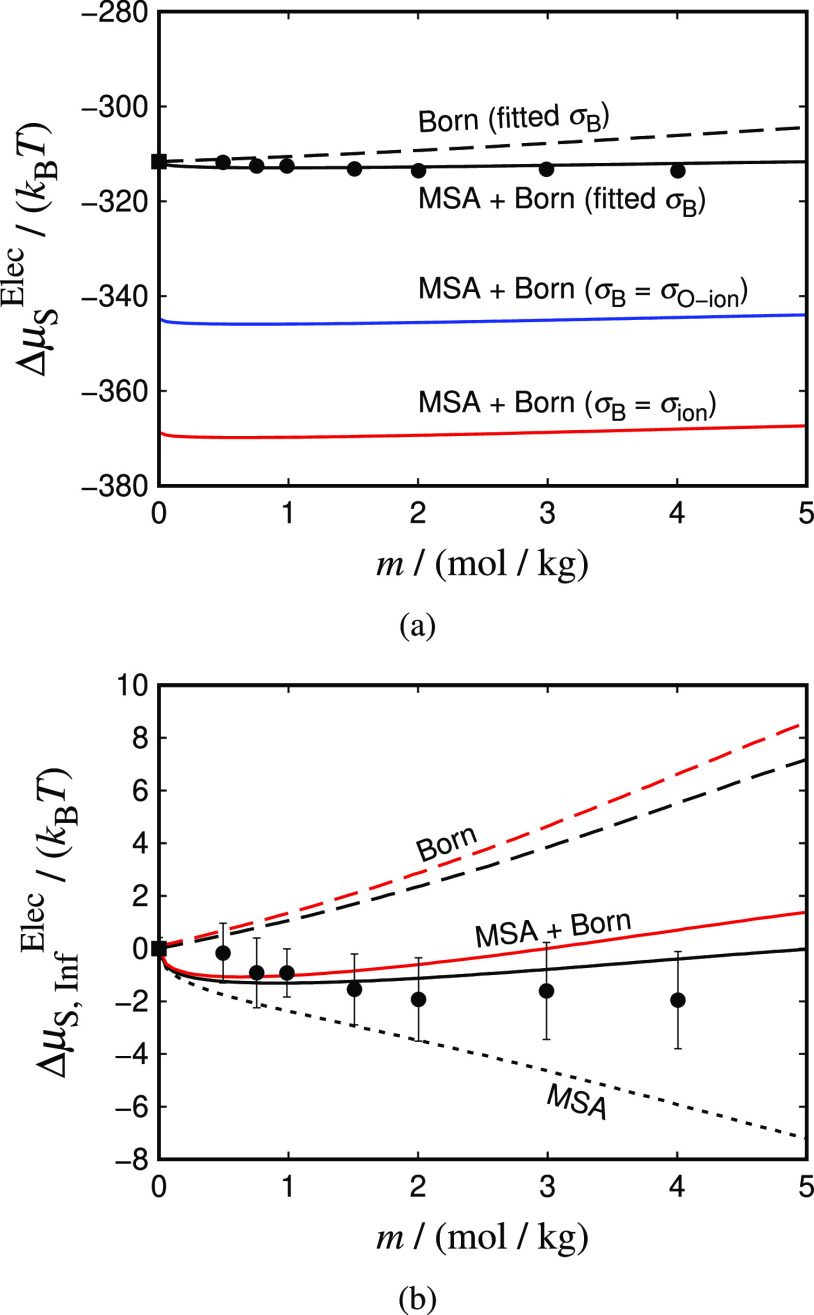
Contributions to the electrostatic chemical potential of aqueous NaCl at 298 K and 1 atm as a function the molality of the system. The chemical potential is shown in absolute value in (a) and in (b) as the rescaled chemical potential obtained by subtracting its value at a reference infinite-dilution state. The symbols represent simulation data obtained with the JC/TIP4P/2005 model.^22^ The impact of the choice of σ_B_ is illustrated in both panels. The red and blue curves represent (respectively) calculations using σ_B_ = σ_ion_ and σ_B_ = σ_O–ion_; the black curves corresponds to calculations obtained using the adjusted value of σ_B_ (see text for details). The continuous curves represent the sum of both the Born and MSA terms, the long-dashed curves correspond to isolated Born calculations, and the short-dashed curve (only shown in (b)) corresponds to the MSA term.

To provide theoretical values for the electrostatic contribution to the chemical potential we assume first that the contact diameter of the ions (Table 2) is also the diameter of the Born cavity to be used in eq 13. The Born contribution to the chemical potential is combined with the ion–ion contribution, obtained using the MSA expression, to calculate the total electrostatic contribution to the chemical potential of the salt. The theoretical calculations are compared with the EE simulation data in Figure 3a, where the calculations can be seen to be in good qualitative agreement with the simulations, although a significant, almost constant, deviation of ∼57*k*_B_*T* is apparent over the molality range considered.

We have shown previously that the MSA theory does not provide accurate quantitative predictions of the ion–ion chemical potential. However, the large difference between the theoretical values and the simulation data for the total electrostatic chemical potential can be mostly assigned to the Born term. The magnitude of the ion–ion contribution (DH or MSA) is of the order of ∼1–2*k*_B_*T* (cf. Figure 1), whereas that of the Born contribution is ∼360*k*_B_*T*. This significant difference in the magnitudes of the Born and ion–ion terms suggests that the former is the dominant contribution to the electrostatic chemical potential, as it determines its magnitude. Moreover, as shown in section 4.1, the MSA and DH theories both lead to overpredictions of the chemical potential, while as can be seen in Figure 3a, the total electrostatic chemical potential is underpredicted by the theoretical calculation.

In order to improve the agreement of the theoretical model with the simulated data, we proceed to fit the value of the Born diameters of the ions using simulation data of the free energy of solvation at 298 K and 1 atm at infinite dilution, since this is the regime where approximations in the Born theory are minimized and, at the same time, there is no contribution from ion–ion interactions. This approach is equivalent to that adopted in recent EoS studies in which the Born diameter is adjusted to achieve a good description of experimental free energies of solvation.^7,10−12^ The EE methodology used to compute the free energy of solvation of the ions is the same as that used to compute the chemical potential of a pair of ions; the only difference is that because individual ions are considered only the corresponding single-ion charge is changed (increased or decreased) in the EE simulation. Because we are simulating a single ion at infinite dilution and we are not considering any kinetic contributions, the Born chemical potential of a given ion is equal to its Born contribution to the free energy of solvation. Molecular simulations of infite dilution free energies have been shown to suffer from finite-size effects^93^ due to the long-range nature of the Coulombic potential and the lack of electrostatic screening by other ions. We have investigated this effect for the system studied here, and estimate that the difference in the salt chemical potential obtained using our chosen system size of 2000 particles compared to that of a larger system of 20 000 particles is less than *k*_B_*T*, which can be considered negligible for the purpose of comparisons with the theoretical calculations.

The free energies of solvation of the anion and cation at infinite dilution are computed by performing EE simulations; we obtain −145.59 *k*_B_*T* for Na^+^ and −166.04 *k*_B_*T* for Cl^–^. These two simulation values are then used to determine new adjusted Born diameters for each of the ions. We choose to use only one data point at infinite dilution for each ion in order to avoid the effects of ion–ion Coulombic interactions. We find that diameters of 0.3779 nm for Na^+^ and 0.3314 nm for Cl^–^ lead to good agreement with the simulation values. These Born diameters are then used to calculate the electrostatic chemical potential of the salt for a range of molalities using MSA to account for the ion–ion interactions and the Born term to include the ion–solvent interactions. The excellent quantitative agreement of the theoretical calculations with the simulation data over the concentration range considered can be seen in Figure 3a.

An important observation from the simulation data presented in Figure 3a is that the electrostatic contribution to the chemical potential of the salt is found to be almost concentration invariant. This is consistent with neutron-diffraction and hydrogen-isotope-substitution experimental observations.^94^ Soper and Weckström^94^ showed that the structure of water is not significantly affected by the presence of ions even up to moderate concentrations. Despite the small magnitude of the calculated theoretical ion–ion chemical potential, the addition of this term is crucial in order to achieve this invariance. We note that the ion term “corrects” the unphysical slope of the Born term, and only the combination of the two terms leads to an invariant chemical potential across different molalities. This can be seen in Figure 3a,b where a calculation of the Born chemical potential of the salt without including the ionic term is seen to lead to a curve with a clear positive slope in concentration.

In Figure 3b, we plot the electrostatic contribution to the rescaled chemical potential (, cf. eq 17) as a function of the molality of the system using the same conditions as in Figure 3a. From this perspective we verify that our conclusion from the previous paragraph still holds; even though the magnitude of the Born and the MSA terms is now comparable, confirming that both terms contribute significantly in the calculation of properties like the osmotic and activity coefficients, the slope of the two terms remains, as expected, opposite. Furthermore, the theoretical calculation that corresponds to the sum of the MSA and Born terms can be seen to be in good agreement with the simulation data (within the statistical uncertainty, which is 2*k*_B_*T*). In addition, a clear improvement in the agreement with the simulation data is seen when the adjusted Born diameter is used in the calculation. This result indicates that the proposed methodology for estimating the Born diameter based on infinite dilution data is a sound approach to developing models for the rescaled chemical potential of solvated ions.

In a recent publication, Simonin^63^ explores the use of the Born theory of solvation in describing the ion–solvent interactions. As mentioned in the introduction, he showed that the magnitude of the Born term is too large when considering rescaled chemical potentials. We do not observe this difference in magnitudes for the case of the JC/TIP4P/2005^22^ model as, both in the case of the fitted σ_B_ and in the case where σ_B_ = σ_ion_, the curves are very close when presented as rescaled chemical potentials. Furthermore, on the basis of ID-MSA calculations, Simonin argued that the ion–solvent contribution to the rescaled chemical potential could exhibit a negative trend, while our simulation results do not corroborate this. However, it should be noted that Simonin found this negative trend only for a single salt (RbCl), which is not studied in our current work.

The adjusted value of the Born diameter for Na^+^ (0.3779 nm) is significantly larger than the value of the ionic diameter in the LJ/TIP4P/2005 force field (σ_Na–Na_ = 0.2160 nm) or the contact diameter of the ion in the NaCl crystal lattice (0.232 nm).^95^ This observation is consistent with models used in EoS studies,^10,11^ where an increase was also seen when comparing the ionic diameter of Na^+^ with the adjusted Born diameter. In the case of Cl^–^ the adjusted Born diameter (0.3314 nm) is found to be significantly smaller than the σ_Cl–Cl_ value (0.4830 nm) in the force field and close to the value of the diameter NaCl crystal lattice (0.334 nm).^95^ In an experimental study, Rashin and Honnig^41^ showed that the Born diameter could be empirically estimated by increasing the ionic lattice diameters by 7%. However, based on our findings in relation to the adjusted Born diameters, it is clear that this empirical rule would not yield satisfactory results. These findings are further discussed in section 4.3, where the structure of the solution is investigated in detail.

**Figure 4:**
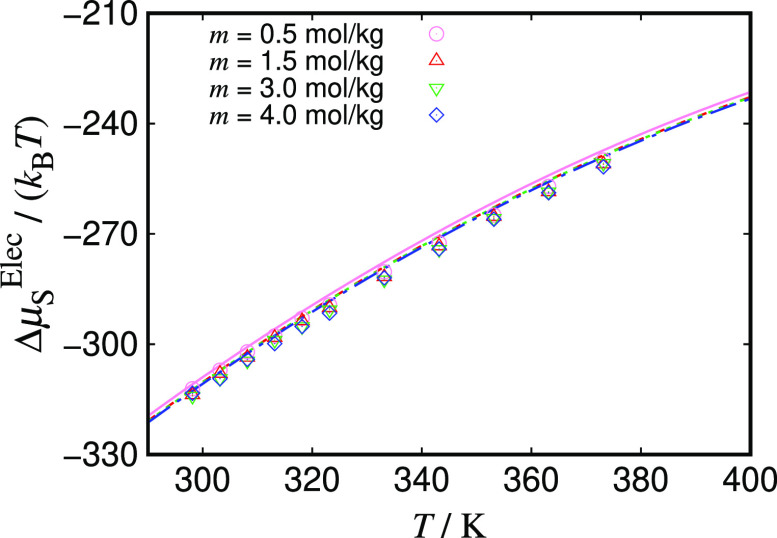
Total electrostatic chemical potential as a function of the temperature of aqueous solutions of NaCl. The symbols represent simulation results obtained using the JC/TIP4P/2005 model,^22^ and the curves represent the sum of the MSA and Born contributions to the chemical potential of the salt, calculated using the adjusted Born diameter (see text for details); the remaining parameters are taken directly from the JC/TIP4P/2005 model.

We proceed to calculate the chemical potential of the salt as a function of temperature at fixed molality using the adjusted Born diameters in the Born term, and compare these calculations with EE simulation data in Figure 4; four values of the molality are considered, to represent the concentration range presented earlier. As before, in order to ensure the best agreement between simulation and theory, values for the dielectric constant as function of temperature to be used in the theoretical expressions are generated using the correlations obtained from the simulated values presented in Figure 2. As the temperature is increased, the electrostatic chemical potential clearly increases. As can be seen in Figure 4, very good agreement between the theoretical calculations for the chemical potential of the salt and the simulations is obtained for the entire range of temperatures considered. The effect of changing the salt concentration on the value of the electrostatic chemical potential of the salt is comparatively very small, at least over the range of concentrations studied. We have already presented this result for calculations at 298 K in Figure 3a; here the same trend is confirmed for temperatures up to 400 K. The excellent agreement between the adjusted model and the simulation data confirms that EoS models where an effective Born diameter for use with the classic Born term provides an accurate description of the ion–solvent electrostatic contribution to the chemical potential across different concentration and temperature ranges.

It is also important to note that although the Born term does not directly depend on the temperature of the solution, an indirect dependence through the choice of a temperature-dependent dielectric constant is introduced in our model. The dielectric constant is determined from the correlations developed using our simulation data, such that the methodology allows us to incorporate essentially exact values of ε_r_ in the theoretical expressions. Any dielectric-constant model that quantitatively reproduces the simulation data would yield comparable results. Here, we choose to use correlations of the simulation data to focus the discussion on benchmarking the Born term as a model for the ion–solvent contribution to the chemical potential of the salt. Although it is gratifying to see the excellent agreement between our proposed model and the simulation data (see Figure 4), it is important to recognize that this result is predicated on the use of an accurate representation of ε_r_, which is therefore of crucial importance. This requirement has been highlighted by several other authors.^24,57,96−98^

**Figure 5:**
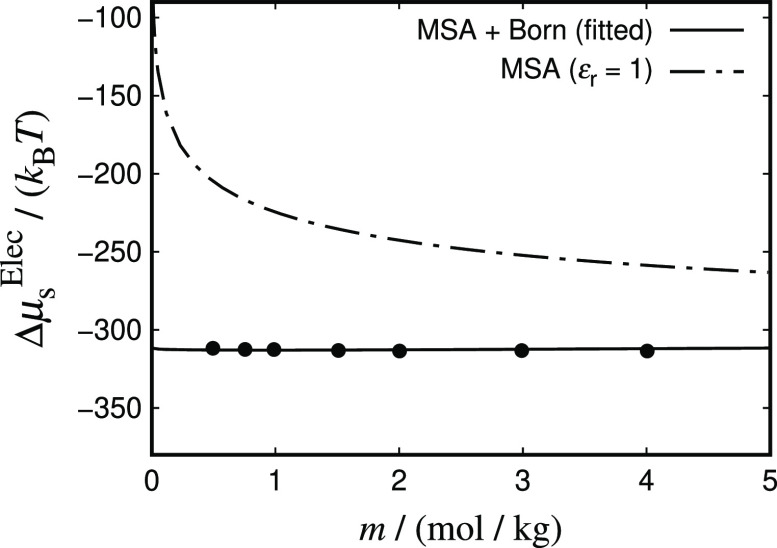
Total electrostatic chemical potential at 298 K and 1 atm as a function of the molality of aqueous solutions of NaCl. The symbols represent simulation results using the JC/TIP4P/2005 model.^22^ Two approaches to modeling the electrostatic chemical potential are compared. The continuous black curve is identical to its counterpart in Figure 3. The dashed−dotted curve represents Δμ_**S**_^Elec^ calculated using MSA with ε_r_ = 1.

At this point, it is useful to note that the theoretical expressions for the ion term have an inverse dependence on the dielectric constant; this leads to the small magnitude of the ion term when considering large dielectric constants. In order to illustrate the importance of using an accurate description of the dielectric-constant, we consider the same states as in Figure 3a, but with the value of the dielectric constant set to its value in vacuum (ε_r_ = 1). Following such an approach implies that the Born term is zero (see eq 13). In Figure 5 we proceed to compare the calculated electrostatic chemical potential using such an approach with the simulation data generated previously. While the calculated electrostatic chemical potential with ε_r_ = 1 does indeed reach a plateau at moderate salt concentrations, when considering low molalities, the qualitative trend is very different from the simulated data. In particular, it is evident that using this approach would make it challenging to model the free energy of solvation, as Δμ^MSA^ tends to zero when no ions are present in solution. Such a result does not mean that it would be impossible to develop accurate theoretical models without incorporating a Born term, but it does imply that the error introduced by such a choice would have to be compensated by other free-energy terms and would lead to unphysical models that deviate significantly from their simulation counterparts.

Through Figures 3a and 5, we have formally shown that it is possible to deliver quantitative agreement for the electrostatic chemical potential of NaCl in water using the MSA and Born expressions. In the current work this is achieved by implementing a quantitatively accurate dielectric constant and a regressed Born diameter. It is especially encouraging to see that the proposed approach is successful even for a sophisticated force field that incorporates the TIP4P/2005 water model.

### 4.3 Fluid Structure

It is useful to compare the radial distribution functions (RDFs) determined with the two force fields considered in the previous sections to investigate the effect of coarse-graining on the local structure of water around the ions and to study ion pairing. As we are principally interested in the electrostatic interactions related to the presence of the ions in solution, we do not present the water–water RDFs here.

**Figure 6:**
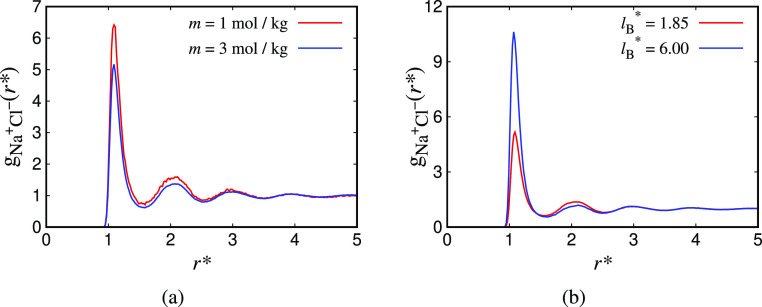
Na–Cl RDFs of the CG/LJ system, where r* = *r*/σ, at T* = 0.75 and ρ* = 0.8. (a) RDFs are computed using *l*_B_^*^=1.85 as initially proposed by Andreev et al.;^78^ (b) the concentration is fixed at *m* = 3 mol/kg and two Bjerrum lengths are compared.

We first consider the CG/LJ model. In Figure 6, the Na–Cl RDFs are shown corresponding to aqueous solutions at two concentrations and at two Bjerrum lengths. As can be seen, the first peak becomes smaller as the salt concentration increases, suggesting that when more ions are added to the mixture, individual ion pairs are driven apart. The reverse trend is observed when the Coulomb interaction is strengthened by increasing the Bjerrum length. Stronger Coulombic interactions result in a sharp increase of the magnitude of the first peak, indicating the formation of loosely bonded ion pairs. Note that the RDF corresponding to *m* = 1 mol/kg is not as smooth as the rest of the curves due to sampling fewer ion pairs overall since the salt concentration is very low at this molality. The corresponding water–water RDF of this model (not shown) remains almost unchanged regardless of the conditions, as the amount of ions present is very small (approximately 5.6% of particles for *m* = 3 mol/kg, and 1.8% for *m* = 1 mol/kg). It is also worth noting that the first water peak has a magnitude of approximately three, which is significantly smaller than the corresponding peaks of the ion–ion RDFs of Figure 6; this is expected, given the strong Coulombic interaction between ions.

In all of the RDFs presented in Figure 6, we observe that a degree of structure persists up to almost 5σ. These long-range correlations result from the long-ranged nature of the Coulomb interactions, which remains in full effect for the CG/LJ model even at long distances, as the lack of an explicitly polar solvent dramatically reduces the screening. We note that the structure obtained is characteristic of that of a LJ liquid state, which is very different from the experimental structure of NaCl in water,^94^ where solvation shells are formed around the ions. Andreev et al.^78^ showed that this model can be used to capture the dynamic properties of aqueous salts qualitatively, but the crude nature of the CG approximation does not make it suitable for structural comparison to experiment.

**Figure 7:**
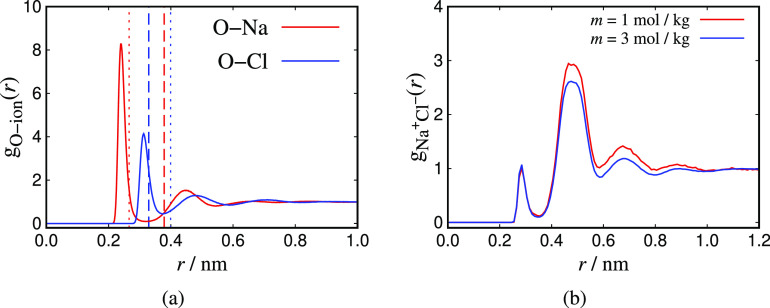
RDFs for the JC/TIP4P/2005 model at 298 K and 1 atm, where *r* is the center–center distance. (a) O–ion RDFs are shown at *m* = 3 mol/kg. Values of *r* corresponding to the estimated Born radii are represented using vertical dashed lines, and those corresponding to the unlike σ are represented using vertical dotted lines. (b) Na–Cl RDF at two different molalities. The density of the solution is kept constant at ρ* = 0.8.

Several authors have related the Born ion diameters to the peaks of the ion–solvent RDFs.^58,99^ Following this approach, we compute the RDF of oxygen–the LJ center of the TIP4P/2005 water–with each of the ions. In Figure 7a, we present the O–Na^+^ and O–Cl^–^ RDFs for an aqueous solution with a molality of 3 mol/kg at *T* = 298.15 K and *P* = 1 atm to keep the conditions the same as in the chemical-potential calculations presented earlier. As can be seen, the first peak of the O–Na^+^ RDF corresponds to the  of the JC/TIP4P/2005 model (Table 2). Interestingly, the first peak of the O–Cl^–^ RDF is found at 0.313 nm, which is significantly smaller than the value of  of the model and is closer to the location of the second peak in the figure. The position of the first peak suggests that extensive overlap occurs in the simulation between the Cl^–^ ions and the water molecules that participate in its solvation shell. This observation supports our earlier results in which the adjusted Born diameters proposed for the anion and cation are found to have values which are specific to each ion. This asymmetry in the adjusted Born diameters of the two ions can be attributed to Cl^–^ extensively overlapping with water.

In models that incorporate the TIP4P/2005 water model, asymmetric overlapping of the ions with the solvent is related to the asymmetric geometry of the water model. In TIP4P/2005 water^79^ the LJ interaction center is placed on the O atom, while the charges are placed off-center. The negative charge of O is placed at the M site, and the H sites are represented only as positive charges without any LJ interaction. As a result, the anions overlap with the water molecules that belong to their solvation shell, since the H atoms do not have a LJ interaction center to repel the ions, but at the same time, the Coulombic force leads to strong attraction between the positive charge of the H and the negative charge of the anions. The extensive overlapping creates solvation shells that are much smaller than the contact  value. This observation is also in agreement with our earlier finding that the regressed parameter for the Born diameter of Cl^–^ is smaller than the O–Cl^–^ contact distance. The decrease in the excluded volume of ions due to the increased ion–solvent Coulombic interaction has been seen in simulation studies (a phenomenon sometimes referred to as electrostriction) and leads to larger solvation energies.^92,100^

In order to assess more details of the molecular structure of the solution, we also present the Na^+^–Cl^–^ RDFs at two salt concentrations. These RDFs are presented in Figure 7b and are in agreement with the corresponding RDFs reported by Benavides et al.^22^ The effect of the existence of the solvation shell can be appreciated here, as the most prominent peak of the RDF does not correspond to . In addition, as the molality of the solution increases, the anion and cation are found to be further apart due to the electric screening caused by the increased concentration of ions. As expected, the Na^+^–Cl^–^ RDF of the JC/TIP4P/2005 model is significantly different to that of the CG/LJ system (cf. Figure 6). The CG water of the CG/LJ force field does not incorporate any charge, and as a result, oriented solvation shells around the ions are not seen.

**Figure 8:**
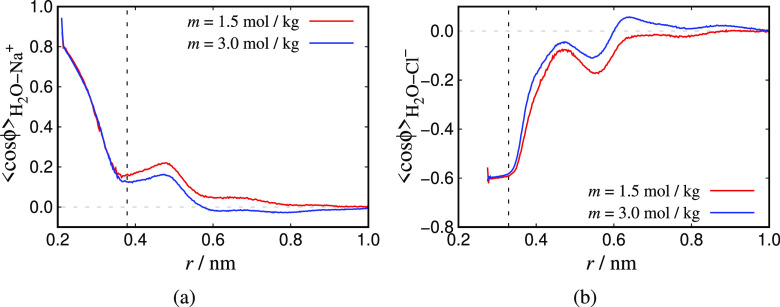
Ion–water ODFs of the JC/TIP4P/2005 model at 298 K and 1 atm. The angular brackets denote ensemble averaged values. Na^+^ is used as the reference (origin) species in (a), and Cl^–^ is used as the reference in (b). The values of *r* corresponding to the estimated Born diameters are shown using vertical dashed lines. The dashed horizontal lines correspond to zero orientational average and are presented in the plots as a guide for the eye.

The local orientation around the ions in the JC/TIP4P/2005 model is depicted in Figure 8 using the ensemble averaged orientational distribution function (ODF) of the angle ϕ between the position (), pointing from the center of a given ion to the LJ center of a water molecule (which is the same as the center of the oxygen), and the dipole moment of the selected water molecule ():
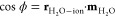
39In practice, the cosine of the angle ϕ is averaged within a spherical shell of radius *r*. The dipole-moment vectors are expected to point toward or (depending on the charge of the ion) outward from the center of the ion which is taken as the origin and, as a result, will be parallel to the distance vector , with a corresponding value of cos ϕ = 1 for Na^+^ and −1 for Cl^–^ as maximum (minimum) values in perfectly aligned configurations of the water dipole–distance vector. In the case of Na^+^ (Figure 8a), at approximately the contact distance  = 0.26590 nm) the water molecules are perfectly oriented around the ion, with a value of the ODF close to 1, and then gradually averages to a random uniform orientational distribution of larger separations. The correlations seen in the ODF correspond to the different geometries of the solvent layers in the solvation shell. In the case of Cl^–^ (Figure 8b), for distances which are smaller than the contact diameter, the average orientation of the water molecules remains roughly constant at a value of –0.6 (corresponding to ∼ 127°). The water molecules are not aligned along their dipole-moment direction, as there are two positive H sites on water, and only one is approaching the ion. This is in agreement with previous reports; an extensive discussion on the structure of water in electrolyte solutions can be found in the experimental work of Soper and Weckström^94^ and the original TIP4P/2005 paper by Abascal and Vega.^79^

In terms of the relationship with the values of ion Born diameters determined in our work, for Na^+^ (Figure 8a), the regressed Born diameter is located approximately at the first minimum of the ODF, while for Cl^–^ (Figure 8b), the regressed Born diameter coincides with the first change in curvature of the ODF, which reflects a significant loss of orientation of the water with respect to the anion. These observations highlight a difference between the anion and the cation in the model that cannot be easily justified, as the Born term does not incorporate any information regarding the orientation of the molecules. However, it is interesting to observe that the asymmetries between the H_2_O–Na^+^ and the H_2_O–Cl^–^ interactions are not limited to the specification of the Born diameters, but also can be related to the different orientational structure around each of the ions.

## 5 Conclusions

A study comparing some of the classic primitive-model expressions for the free energy of electrostatic interactions in electrolyte solutions with simulation data is presented.

The electrostatic contribution to the chemical potential obtained with the MSA and DH theories are tested against EE simulation data using the nonpolar CG model of water of Andreev et al.^78^ The MSA and DH theories are found to be of similar accuracy for this model system, with the MSA approach resulting in marginally better agreement with the simulations. In principle, models for which the ion–ion contribution is important could see an improved accuracy if the MSA expressions are used instead of the DH approach. An example for which one might anticipate such an improvement are EoS models that account for electrostatic interactions only between ions and treat electrostatic interactions between the solvent and the ions as dispersion interactions.^4,8,53^

In addition, a combination of the MSA approach and the Born theory of solvation is used to calculate the electrostatic contribution to the chemical potential of NaCl in TIP4P/2005 water using the model parameters of Benavides et al.^22^ It is found that by adjusting the Born diameter to achieve agreement between the theoretical and simulated infinite dilution free energy of solvation for Na^+^ and Cl^–^ at 298 K and 1 atm, one can achieve an excellent quantitative description of the chemical potential of the salt across a broad range of concentrations and temperatures. The temperature dependence of the Born theory of solvation is incorporated into the theory using *NVT* simulation data of the dielectric constant of the model. The importance of implementing a good quantitative model for this property of the solution is highlighted. Although the ion–ion contribution to the free energy is found to be 2 orders of magnitude smaller than that of the ion–solvent contribution, a combination of both the MSA and Born theory terms is essential to deliver values of the chemical potential of the ionic species in the solution that are in quantitative agreement with the simulations.

As well as the residual contributions to the chemical potential, we have also investigated the contribution of the Born and MSA terms to the rescaled chemical potential relative to its value at infinite dilution. This rescaled representation of the chemical potential is important as it is used in activity coefficient calculations. In this context, we observe that adjusting the Born diameter of the ions leads to better agreement with simulation data. Nevertheless, it should be noted that using the diameter of the ions as the Born diameter also yields results that exhibit good qualitative behavior.

An alternative approach for comparison with the simulation data is also considered in which the vacuum dielectric constant ε_r_ = 1 is used, such that only the ionic term of the free energy (treated in the MSA approach) remains as an electrostatic contribution (the Born term cancels for ε_r_ = 1). Although for moderate salt concentrations the qualitative behavior of such model follows the trend of the simulation data, at low concentrations the model does not deliver the correct infinite-dilution behavior that is essential for the description of the solvation free energy. This illustrates the importance of incorporating the solvent–ion interactions using the Born theory in primitive models, as well as the critical importance of incorporating a quantitatively accurate dielectric constant in the models.^101^

The RDF and ODF of the solution are investigated to complement the discussion. Of the two models simulated, the nonpolar CG water is shown not to form oriented solvation shells around the ions, and instead exhibits a standard LJ liquid structure. When the refined Born diameters are compared to the fluid structure of the JC/TIP4P/2005 model, no correlation is found with the RDF. By contrast, the changes in local orientation within the solvation shells around the ions are shown to correlate with these regressed parameters.

The current work is the first study in which, using simulation data for comparison, the MSA, DH, and Born theories for electrolyte solutions are assessed as perturbation approaches for use in the context of equations of state. This is achieved by isolating the electrostatic contributions and comparing each term with simulation data generated without contributions from dispersion interactions. In the case of the JC/TIP4P/2005 model,^22^ these simulations are especially demanding as the free-energy difference between the charged and the uncharged states of the test particles is very large, with little overlap in their probable configurations.

We expect the findings from our study to be pertinent in two different and topical areas of research in electrolyte-solution thermodynamics. First, benchmarking the various theoretical contributions to the chemical potential of the salt using simulation data will facilitate future studies in which EoS-type approaches for electrolyte solutions are assessed. Second, establishing a promising and robust methodology to develop quantitatively accurate EoS will be invaluable in guiding future development of thermodynamic models of electrolyte solutions. Extending the conclusions of the current work to divalent, multivalent, and mixed salt electrolytes, for example, would be especially relevant.

## Supporting Information Available

Expressions for the calculation of the dielectric constant correlated to the data of Figure 2 (PDF)
