# Supplementary material for: The Contribution of the Ion–Ion and Ion–Solvent Interactions in a Molecular Thermodynamic Treatment of Electrolyte Solutions
Source: J Phys Chem B. 2022 Nov 17;126(47):9821–39. doi: 10.1021/acs.jpcb.2c03915 (PMC9720728; doi:10.1021/acs.jpcb.2c03915)
Supplement: Supplementary file 1 — jp2c03915_si_001.pdf [file jp2c03915_si_001.pdf]

Supplementary Information: The contribution of the ion–ion and ion–solvent interactions in a molecular-thermodynamic treatment of electrolyte solutions

Spiros Kournopoulos,[†] Mirella Simões Santos,[‡] Srikanth Ravipati,[†] Andrew J. Haslam,[†] George Jackson,[†] Ioannis G. Economou,[¶] and Amparo Galindo[*,†]

†Department of Chemical Engineering, Sargent Centre for Process Systems Engineering, and Institute for Molecular Science and Engineering, Imperial College, London, London SW7 2AZ, United Kingdom

‡Laboratoire de Chimie, École Normale Supérieure de Lyon, 46 Allée d'Italie, 69364, Lyon, France; and Australian Institute for Bioengineering and Nanotechnology, The University of Queensland, Brisbane, Australia

¶Chemical Engineering Program, Texas A&M University at Qatar, Doha, Qatar

E-mail: a.galindo@imperial.ac.uk

Here we provide the correlated expressions to the data of Figure 2 of the manuscript:

$$\varepsilon_{\mathrm{r}} = 0.6165m^2 - 9.1826m + 55;$$

$$\varepsilon_{\mathrm{r}} = -0.1571T + 98.876;$$

$$\varepsilon_{\mathrm{r}} = -0.1398T + 85.216;$$

$$\varepsilon_{\mathrm{r}} = -0.0886T + 60.323;$$

$$\varepsilon_{\mathrm{r}} = -0.0953T + 57.786.$$

The quadratic expression resulting from correlating the data in figure 2a is shown first, followed by the linear correlations obtained from the data in figure 2b (top to bottom). We remind the reader that ε_{r} denotes the dielectric constant, m the molality of the solution in mol / kg, and T the temperature in K.
